# Exogenous surfactant prevents hyperoxia-induced lung injury in adult mice

**DOI:** 10.1186/s40635-019-0233-6

**Published:** 2019-03-27

**Authors:** Frank Silva Bezerra, Camila de Oliveira Ramos, Thalles de Freitas Castro, Natália Pereira da Silva Araújo, Ana Beatriz Farias de Souza, Ana Carla Balthar Bandeira, Guilherme de Paula Costa, Christiane Teixeira Cartelle, André Talvani, Sílvia Dantas Cangussú, Laurent Brochard, Akinori Cardozo Nagato

**Affiliations:** 1 Laboratory of Experimental Pathophysiology (LAFEx), Department of Biological Sciences (DECBI), Center of Research in Biological Sciences (NUPEB), Federal University of Ouro Preto (UFOP), Ouro Preto, Brazil; 2 Laboratory of Immunobiology of Inflammation (LABIIN), Department of Biological Sciences (DECBI), Center of Research in Biological Sciences (NUPEB), Federal University of Ouro Preto (UFOP), Ouro Preto, Brazil; 3 Laboratory of Neuro Immuno experimental pathology (NIPE), Department of Pathology, Institute of Biological Sciences, Federal University of Minas Gerais (UFMG), Belo Horizonte, Brazil; 4 Laboratory of Immunopathology and Experimental Pathology, Center for Reproductive Biology—CRB, Federal University of Juiz de Fora, Juiz de Fora, Minas Gerais Brazil; 5 Physiology Department, Federal University of Juiz de Fora, Juiz de Fora, Minas Gerais Brazil; 6 Keenan Research Centre, Li Ka Shing Knowledge Institute, St. Michael’s Hospital, Toronto, ON Canada; 7 Interdepartmental Division of Critical Care Medicine, University of Toronto, Toronto, ON Canada; 8 Laboratory of Experimental Pathophysiology (LAFEx), Department of Biological Sciences (DECBI), Institute of exact and biological sciences (ICEB), Federal University of Ouro Preto (UFOP), Campus Universitário s/n, Morro do Cruzeiro, Ouro Preto, MG 35400-000 Brazil

**Keywords:** ARDS, BALB/c mice, Exogenous surfactant, Hyperoxia, Lung injury, Oxidative stress

## Abstract

**Background:**

In addition to the risk of developing ventilator-induced lung injury, patients with ARDS are at risk of developing hyperoxic injury due the supra-physiological oxygen supplementation clinically required to reverse hypoxemia. Alterations of endogenous surfactant system participate in the pulmonary dysfunction observed in ARDS. Administration of exogenous surfactant could have protective effects during hyperoxia.

**Methods:**

Male BALB/c mice (8–10 weeks), a strain highly sensitive to hyperoxia, received the exogenous surfactant-containing protein SP-B and SP-C by intranasal instillation 12 h before starting 24 h of exposure to hyperoxia in an inhalation chamber and were compared to mice receiving hyperoxia alone and to controls subjected to normoxia.

**Results:**

Compared to the hyperoxia group, the administration of exogenous surfactant was able to reduce lung inflammation through a reduction in the influx of neutrophils and inflammatory biomarkers such as TNF, IL-17, and HMGB1 expression. The antioxidant activity prevented oxidative damage by reducing lipid peroxidation and protein carbonylation and increasing superoxide dismutase activity when compared to the hyperoxia group.

**Conclusion:**

Our results offer new perspectives on the effects and the mechanism of exogenous surfactant in protecting the airway and lungs, in oxygen-rich lung microenvironment, against oxidative damage and aggravation of acute inflammation induced by hyperoxia.

## Background

Studies have shown that alterations of the endogenous surfactant system contribute to pulmonary dysfunction and atelectasis in ARDS [1]. These alterations include decrease of pool size and functional modifications of endogenous surfactant. Mechanical ventilation by itself can lead to the oxidation of surfactant components [1]. A meta-analysis of studies in adult patients with ARDS showed that exogenous surfactant (ES) administration may improve oxygenation in the first 24 h but does not mitigate mortality and long-term oxygenation (> 120 h) [2]. Important concerns have been raised, however, regarding the practical administration of surfactant and its ability to reach the alveoli [3]. Recently, our research team showed that ES administration decreased oxidative stress in the lungs of mice exposed to cigarette smoke [4].

Hyperoxia can promote changes in the function of surfactant subtypes [5] and is able to induce inflammation and oxidative stress [6]. In humans, an administration of high concentrations of oxygen has shown that the lung is susceptible to oxygen toxicity after breathing pure oxygen for only 17 h [7]. Several studies have demonstrated a higher risk of mortality in patients treated with high concentrations of oxygen, but the mechanisms are unclear [8–11]. Several studies using knock-out mice showed that deficiencies in several surfactant proteins (SP-A, SP-B, SP-C, and SP-D) result in higher inflammation induced by hyperoxia [12–14] and upregulation of cytokines, such as the high mobility group box protein 1 (HMGB1) [15]—a pro-inflammatory cytokine expressed by lung epithelial and endothelial cells and alveolar macrophages [16], and which attracts neutrophils, releases cytokines as TNFα, and inhibits macrophages migration [17]. SP-D can also inhibit the secretion of TNF, IFNγ, and IL-6 [18]; decreases peroxidation of surfactant lipid mixtures [19]; and reduces recruitment of inflammatory cells to lungs after infection [20], while SP-A inhibits IL-10 production and decreases recruitment of macrophages [21]. It has been shown that the inhibition of extracellular HMGB1 attenuated hyperoxia-induced inflammatory acute lung injury followed by reduction of leukocyte and polymorphonuclear cells [16], while other experimental models confirm that the depletion of HMGB1 reduces acute lung injury [22, 23]. None of these experimental models, however, was originally designed to investigate in vivo the combined effects of surfactant on hyperoxia-induced inflammation.

This study evaluated the effect of exogenous surfactant administration to prevent hyperoxic acute lung injury in BALB/c adult mice. This mice strain was used due to its sensitivity to hyperoxia [6].

## Methods

### Animals

Male BALB/c mice (8–10 weeks, 20–25 g) were purchased from the Animal Science Center (ASC) of the Federal University of Ouro Preto (Ouro Preto, MG, Brazil) in a controlled-environment with cycled lighting (12 h light/12 h dark, lights on at 6:00 PM), with controlled temperature (21–22± 2 °C) and relative humidity (50 ± 10%). The animals received food and water ad libitum. The experimental design was approved by the Ethics Committee for Animal Research of UFOP (No. 2015/14).

The animals were divided into four groups: Control group (CG)—control mouse was exposed to normoxia in air room. Surfactant group (SG)—mice were challenged with ES by intranasal instillation 12 h before hyperoxia. Hyperoxia group (HG)—the animals were exposed to 100% oxygen in a chamber for 24 h. Hyperoxia-surfactant group (HSG)—mice were challenged with ES by intranasal instillation 12 h before hyperoxia followed by exposure to 100% oxygen in a chamber for 24 h.

### Hyperoxia protocol

A cylinder containing 8000 L (180 Kgf/cm^2^, White Martins, Praxair Inc., São Paulo, Brazil) medical O_2_ was coupled to the *Bourdon* tube and the *Thorpe* tube (0–15 L / min.). A silicone conduit was connected to the Thorpe tube and the oxygen inhalation chamber (20x15x30cm), as described previously [6]. At the end of the oxygen exposure, the animals were euthanized by an overdose of ketamine (130 mg/kg) and xylazine (0.3 mg/kg).

### Surfactant administration

The ES (The CUROSURF brand surfactant (Chiesi Farmaceutici S.p.A., Parma, Italy) was administered by intranasal instillation 12 h before exposure to hyperoxia in only one dose of 2.0 mL/kg/day (50 μL) [4].

### Bronchoalveolar lavage fluid

The thorax of each animal was opened for collection of the bronchoalveolar lavage fluid (BALF). The left lung was clamped, the trachea was cannulated and the right lung was washed with 3 × 500 μL of saline solution. A 250 μL/sample was centrifuged at 1000 rpm for 1 min (cytospin technique—*g* Force). The total count of cells in the BALF was performed using a Neubauer chamber and the differential count using Panotic stained with Fast (Laborclin, Paraná, BR). The identification of inflammatory cells and differential counts was performed on slides by light microscopy [24].

### Tissue processing and homogenization

After BALF collection, the right ventricle of each animal was perfused with saline to remove blood from the lungs. The right lung was clamped so that only the left lung was perfused with buffered 4% formalin (pH 7.2) via the trachea. The material was then processed and stained with hematoxylin and eosin for histological analysis. The right lung was removed and stored in labeled tubes. Afterwards, the right lung was homogenized in phosphate buffer (pH 7.5) and centrifuged at 10.000 rpm for 10 min. Supernatant was collected, and the samples were stored (− 80 °C) for biochemical analyses.

### Immunoassays for inflammatory markers in BALF

BALF were used for the analyses of TNF, IL-17, and CCL5. Immunoassays (Peprotech kits, Ribeirão Preto, Brazil) were performed in 96-well plates on which 100 μl of monoclonal antibody to the protein (or peptide) of interest was added and samples were diluted in PBS containing 0.1% bovine serum albumin-BSA (Sigma-Aldrich, St Louis, MO). After incubation for 12 h at room temperature, the plates were blocked with 300 μl/well of a PBS solution containing 1% BSA for 1 h at 37 °C. Samples were applied in a volume of 25 μl to each well. The reaction was read on ELISA reader at 490 nm.

### Antioxidant enzymes and biomarkers of oxidative damage

All chemicals were purchased from Sigma-Aldrich Chemical Co., (Sigma-Aldrich Inc., St. Louis, MO, USA). All measurements described below were performed on lung homogenates using a spectrophotometer (Beckman model DU 640; Fullerton, CA) or a microplate reader (Bio-Rad model 550, Hercules, CA). Catalase (CAT) activity was calculated from the rate of decrease in the concentration of hydrogen peroxide (U/mg protein), which was determined from the absorbance at 240 nm. The SOD activity was measured according to the Marklund method [25] which is based on the ability of the enzyme to inhibit the auto-oxidation of pyrogallol. Absorbances were read on ELISA reader at a wavelength of 570 nm. The oxidative damage was determined by levels of malondialdehyde (MDA) measured during an acid-heating reaction with thiobarbituric acid and was determined from the absorbance at 535 nm (described by Buege et al.) [26]. The carbonylation of proteins was performed (according to Levine et al. [27]). The total protein analysis was performed by the Bradford method [28].

### Morphometric stereological analysis

The volume density analyses of the alveolar septum (Vv [sa]) and the alveolar spaces (Vv [a]) were performed in a test system composed of 16 points and a known test area. The test system was coupled to a monitor attached to a microscope. The number of points (Pp) that reached the alveolar septa (Vv [sa]) and the alveolar spaces (Vv [a]) were evaluated according to the total number of points in a test system (Pt). The reference volume was estimated by the point-of-use counting of the test point (Pt) systems. A total area of 1.94 mm^2^ was analyzed to determine the volume densities of the alveolar septa (Vv [sa]) and the alveolar spaces (Vv [a]) in sections stained with H&E, respectively [24, 29].

### Immunohistochemical assay

Histological sections were stained with HMGB1 (EPR3507) (Abcam, UK) by immunohistochemistry. Morphometric analysis of the sections stained by immunohistochemistry was performed in 20 random fields of the slides photographed at a magnification of × 20 using ImageJ 1.6.0 software. In each field, the total number of nuclei and the number of nuclei labeled for the antibody used were counted, and the ratio of labeled nuclei/total nuclei was calculated [30].

### Blood collection

A blood aliquot of each animal was collected by means of cardiac puncture in polypropylene tubes with 15 μL of anticoagulants to evaluate hematological parameters. Bc2800vet Auto Hematology Analyzer (Mindray® Bio-Medical Electronics Co. Ltda, Shenzhen, China) was used for the analyses. Futhermore, to perform the blood smear, 2 μL of blood was pipetted (automatic pipette) at one end of a sterile histological slide. They were stained with the Quick Panoptic Kit (Laborclin, Pinhais, Paraná, Brazil). A total of 100 leukocytes were counted per slide, which were differentiated into monocytes, neutrophils, and lymphocytes [31].

### Statistical analysis

The sample size was calculated using a statistical power of 95% and a level of significance of 5% (BioEstat 5.3). The variable used to calculate power was alveolar macrophages, we were looking for 30% reduction. Data were expressed as mean ± SD. Evaluation of data normality was performed using the Kolmogorov-Smirnov test. The univariate analysis of variance (ANOVA one-way) followed by Newman-Keuls post-test was used for parametric data. The Kruskal-Wallis test followed by Dunn’s post-test was used for nonparametric data. A significant difference was considered when *p* < 0.05. All analyses were performed using GraphPad Prism software version 5.00 for Windows 7, GraphPad Software (San Diego, CA, USA).

## Results

### Total and differential leukocyte count in BALF

Hyperoxia promoted a reduction of total leukocytes (ANOVA; *P* < 0.0001) and macrophages (ANOVA; *P* < 0.0001) compared to control and surfactant groups (*p* < 0.05), though the absolute levels of neutrophils (ANOVA; *P* = 0.0068) were significantly higher than in control group animals (*p* < 0.05). The exogeneous surfactant administration decreased the levels of neutrophils and increased the levels of macrophages in animals exposed to hyperoxia (*p* < 0.05). Lymphocytes were not different than from the control group. By comparison to the control, surfactant alone did not induce any change in the level of inflammatory cells (Table 1).

**Table 1:** Inflammatory cell in bronchoalveolar lavage

Group	CG *n* = 9	SG *n* = 9	HG *n* = 9	HSG *n* = 9
Leucocytes (× 10^3^/mL)	97.07 ± 13.66	93.72 ± 10.48	64.99 ± 10.48 ^a,b^	71.65 ± 13.66^a,b^
Macrophages (× 10^3^/mL)	94.23 ± 13.33	87.86 ± 13.33	48.35 ± 6.39 ^a,b^	66.96 ± 11.06^c^
Lymphocytes (× 10^3^/mL)	2.28 ± 1.06	1.63 ± 2.04	2.48 ± 1.20	2.16 ± 2.20
Neutrophils (× 10^3^/mL)	0.56 ± 0.54	4.23 ± 3.14	14.16 ± 11.45 ^a,b^	2.53 ± 2.18^c^

^a^Control group (CG)—control mouse exposed to normoxia in air room, without surfactant. Surfactant group (SG)—mice were challenged with exogenous surfactant (surfactant challenge 2.0 mL/kg/day) by intranasal instillation (12 h before hyperoxia). Hyperoxia group (HG)—the animals were exposed to 100% oxygen in chamber for 24 h. For more details, see the “Methods” section. Hyperoxia-surfactant group (HSG)—mice were challenged with exogenous surfactant (surfactant challenge - 2.0 mL/kg/day) by intranasal instillation (12 h before hyperoxia) after exposure lungs to 100% oxygen in chamber for 24 h. significant difference between the groups when compared to the CG; ^b^significant difference between the groups when compared to the SG; ^c^significant difference between the groups when compared to the HG. Data are expressed as mean ± SD and were analyzed by one-way ANOVA followed by Newman-Keuls’s post-test (*p* < 0.05)

### Inflammatory markers in BALF

The levels of TNF, CCL5 and IL-17 in BALF were analyzed to assess the effects of exogeneous surfactant administration. Hyperoxia resulted in an increase in the levels of TNF (ANOVA; *p* = 0.0003) and IL-17 (ANOVA; *p* = 0.0006) when compared to control and surfactant groups (*p* < 0.05). These levels decreased with the administration of surfactant compared to hyperoxia group (*p* < 0.05). In addition, the exposure to hyperoxia resulted in a decrease of CCL5 (ANOVA; *p* = 0.0028) compared to control and surfactant group (*p* < 0.05). Surfactant administered before hyperoxia was not able to restore the levels of CCL5 compared to hyperoxia group. By comparison to control, surfactant alone did not induce any change in inflammatory markers in BALF (Table 2).

**Table 2:** Biochemical analysis of damage and oxidative stress and inflammatory cytokines in lung tissue homogenates

Group	CG *n* = 9	SG *n* = 9	HG *n* = 9	HSG *n* = 9
SOD (U/mg ptn)	84.25 ± 7.68	76.26 ± 8.70	59.47 ± 5.25 ^a^	93.63 ± 23.43^c^
CAT (U/mg ptn)	1.45 ± 0.46	1.09 ± 0.24	1.26 ± 0.18	1.57 ± 0.39
TBARS (nmol/mg ptn)	1.63 ± 0.15	1.72 ± 0.15	2.04 ± 0.24^a,b^	1.47 ± 0.44^c^
Protein carbonyl (nmol/mg ptn)	18.15 ± 1.91	15.58 ± 1.97	25.34 ± 1.86^a,b^	18.01 ± 5.08^c^
TNFα (pg/mL)	154.5 ± 8.21	156.6 ± 10.98	226.0 ± 33.96 ^a,b^	152.5 ± 34.47^c^
CCL5 (pg/mL)	351.5 ± 68.68	345.4 ± 58.46	259.2 ± 45.63 ^a,b^	221.9 ± 61.08 ^a,c^
IL-17 (pg/mL)	351.9 ± 45.78	352.6 ± 148.70	616.0 ± 84.07 ^a,b^	333.2 ± 77.31 ^c^

Control group (CG)—control mouse exposed to normoxia in air room, without surfactant. Surfactant group (SG)—mice were challenged with exogenous surfactant (surfactant challenge 2.0 mL/kg/day) by intranasal instillation (12 h before hyperoxia). Hyperoxia group (HG)—the animals were exposed to 100% oxygen in chamber for 24 h. For more details, see the “Methods” section. Hyperoxia-surfactant group (HSG)—mice were challenged with exogenous surfactant (surfactant challenge 2.0 mL/kg/day) by intranasal instillation (12 h before hyperoxia) after exposure lungs to 100% oxygen in chamber for 24 h. *SOD* superoxide dismutase, *CAT* catalase, *TBARS* thiobarbituric acid reactive substances, *TNF* tumor necrosis factor, *CCL5* C-C motif chemokine ligand 5 (RANTES), *IL-17* Interleukin-17. ^a^significant difference between the groups when compared to the CG; ^b^significant difference between the groups when compared to the SG; ^c^significant difference between the groups when compared to the HG. Data are expressed as mean ± SD and were analyzed by one-way ANOVA followed by Newman-Keuls’s post-test (*p* < 0.05)

### Redox status analyses

The analysis of lung homogenate revealed important protective effects of the surfactant on hyperoxia-induced pulmonary damage. The exposure to 100% oxygen resulted in increased levels of the malondialdehyde (ANOVA; *p* = 0.0143) and protein carbonyl content (ANOVA; *p* = 0.0167) when compared to control and surfactant groups (*p* < 0.05). The levels of malondialdehyde and protein carbonyl were reduced compared to animals exposed to hyperoxia (*p* < 0.05). The SOD activity (ANOVA; *p* = 0.0125) decreased in the presence of 100% oxygen but increased with administration of exogenous surfactant (*p* < 0.05). Regarding CAT activity (ANOVA; *p* = 0.1750), there were no difference among the experimental groups. By comparison to control, surfactant alone did not induce any change in relation the redox status in lung homogenate in our experimental model of exposed to hyperoxia (Table 2).

### Morphometric analyses of pulmonary parenchyma

**Fig. 1:**
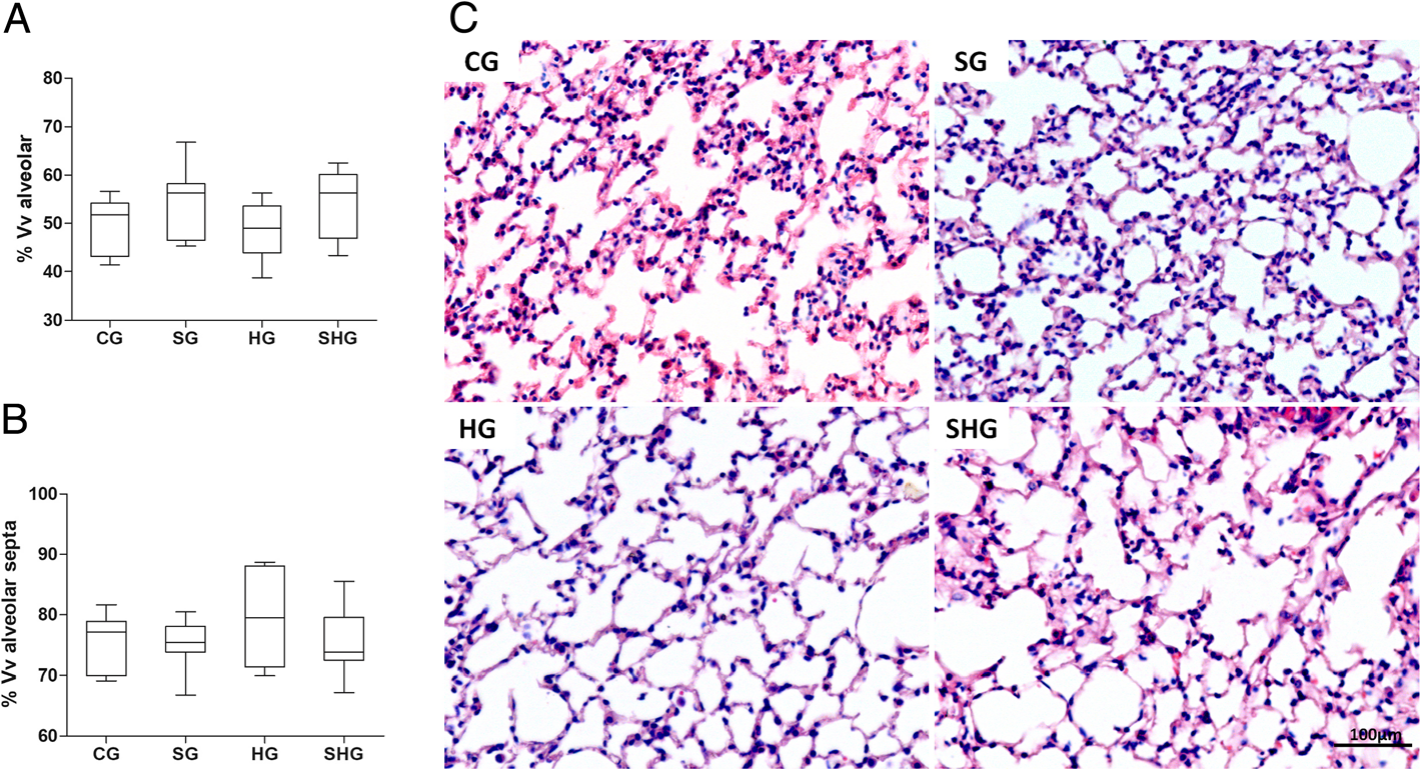
Stereological analyses of lung sections. **a** Volume density of alveolar septa and **b** volume density of alveolar airspace. **c** Histological section of lung parenchyma stained with HE. Barr = 50 μm. CG control group, SG surfactant group, HG hyperoxia group, HSG hyperoxia and surfactant group

**Fig. 2:**
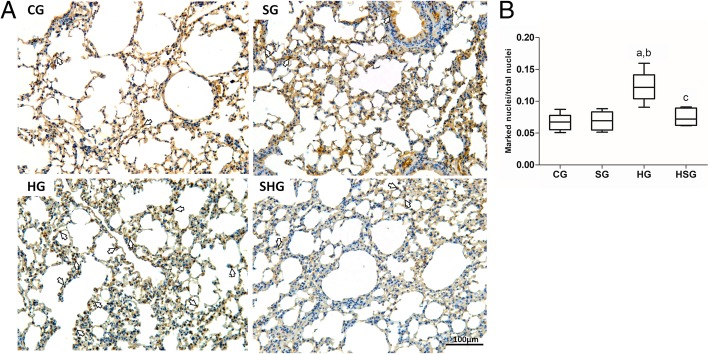
Immunohistochemistry for HMGB1. **a** Histological section of lung parenchyma stained by immunohistochemical technique. Barr = 100 μm. The arrows point to marked nuclei. **b** Ratio between the number of nuclei labeled for HMGB1 antibody and the total number of nuclei. Data are expressed as mean ± SD (*n* = 9). (a) represents a significant difference between the groups when compared to the CG; the letter (b) is significant difference between the groups when compared to the SG, and letter (c) represents a significant difference between the groups when compared to the HG. Data were analyzed by one-way ANOVA followed by Newman-Keuls’s post-test

The stereological analyses showed no significant difference among groups for Vv [a] (ANOVA; *p* = 0.1994) and Vv [sa] (ANOVA; *P* = 0.4955) as observed in Fig. 1. We investigated the expression of HMGB1 in lung parenchyma by immunohistochemistry. Alone, hyperoxia led to a significant increase of HMGB1 expression (ANOVA; *P* = 0.0013) in lung parenchyma compared to control and surfactant groups (*p* < 0.05). The administration of exogenous surfactant was able to reduce the levels of HMGB1 in lung parenchyma of animals exposed to hyperoxia (p < 0.05). By comparison to control, surfactant alone did not induce any change the levels of HMBG1 (Fig. 2).

### Hematological parameters

The exposure to hyperoxia increased the number of erythrocytes (ANOVA; *p* = 0.0024), hematocrit (ANOVA; *p* = 0.0061), hemoglobin (ANOVA; *p* = 0.0127) when compared to control and surfactant groups (*p* < 0.05), while surfactant administered before hyperoxia prevented most of the effects when compared to hyperoxia group (*p* < 0.05). There was a decrease in leucocytes (ANOVA; *p* = 0.0044) when compared to control and surfactant groups (*p* < 0.05). Monocytes (ANOVA; *p* = 0.0374) and neutrophils (ANOVA; *p* < 0.0001) were lower in animals exposed to hyperoxia than in the control group (p < 0.05). Lymphocytes were not different than from control group (Table 3).

**Table 3:** Hematologic parameters in BALB/c mice after hyperoxia and/or exogenous surfactant

Group	CG *n* = 9	SG *n* = 9	HG *n* = 9	HSG *n* = 9
Erythrocyte (× 10^6^/mm^3^)	7.70 ± 0.65	7.80 ± 0.60	9.09 ± 0.30^a,b^	8.13 ± 0.42^c^
Hematrocit (%)	37.08 ± 3.89	37.68 ± 2.66	44.34 ± 1.19 ^a,b^	39.90 ± 1.66^c^
Hemoglobin (g/dL)	12.36 ± 1.56	12.56 ± 0.91	14.78 ± 0.59 ^a,b^	13.30 ± 1.66^c^
Leucocytes (× 10^3^/mL)	4.07 ± 0.93	4.05 ± 0.53	2.55 ± 0.48 ^a,b^	3.04 ± 0.60
Monocyte (×10^3^/mL)	0.85 ± 0.40	0.75 ± 0.18	0.37 ± 0.08 ^a^	0.73 ± 0.19
Lymphocytes (× 10^3^/mL)	2.55 ± 0.85	3.08 ± 0.73	2.12 ± 0.37	2.26 ± 0.43
Neutrophils (× 10^3^/mL)	0.67 ± 0.27	0.22 ± 0.17 ^a^	0.06 ± 0.02 ^a^	0.05 ± 0.01 ^a^

Control group (CG)—control mouse exposed to normoxia in air room, without surfactant. Surfactant group (SG)—mice were challenged with exogenous surfactant (surfactant challenge 2.0 mL/kg/day) by intranasal instillation (12 h before hyperoxia). Hyperoxia group (HG)—the animals were exposed to 100% oxygen in chamber for 24 h. For more details, see the “Methods” section. Hyperoxia-surfactant group (HSG)—mice were challenged with exogenous surfactant (surfactant challenge 2.0 mL/kg/day) by intranasal instillation (12 h before hyperoxia) after exposure lungs to 100% oxygen in chamber for 24 h. ^a^significant difference between the groups when compared to the CG; ^b^significant difference between the groups when compared to the SG; ^c^significant difference between the groups when compared to the HG. Data are expressed as mean ± SD and were analyzed by one-way ANOVA followed by Newman-Keuls’s post-test (*p* < 0.05)

## Discussion

In this study, we evaluated the preventive effects of exogenous surfactant administration on an experimental model of exposure to pure oxygen**.** Our results showed that administration of exogenous surfactant was able to reduce lung inflammation through the reduction of inflammatory biomarkers such as TNF and HMGB1, as well as antioxidant activity by reducing the redox imbalance caused by exposure to hyperoxia. In regards to hyperoxia, our results show similar levels of BAL inflammatory cells and TNF which have been previously associated with lung tissue injury (based on MMP2, MMP9, and inflammatory scores) in studies from our group [6].

### Immunomodulatory and anti-inflammatory effect of exogenous surfactant exposure prior to hyperoxia

In the present study, we observed a lower number of monocyte and macrophages in hyperoxia conditions with concomitant low expression of CCL5, possibly because hyperoxia-induced apoptosis of alveolar macrophages (as *in culture*) mediated by mitogen-activated protein kinase pathway [32].

Macrophages are important producers of CCL5 [33], and the exposure to hyperoxia leads to programmed death of the alveolar macrophages. The absence of alveolar macrophages activated seems to polarize immune response toward T cells and dendritic cells [34].

In the present study, high levels of neutrophils were attracted into the lung, probably resulting from a chemoattraction guided by HMGB1-modulated IL-17. This could have important influence on oxidative damage, because neutrophils are a great source of reactive oxygen species [35]. In a previous study, a concomitant elevation of IL-17 levels and neutrophils in the airways induced by hyperoxia were observed [36]. A precursor study showed that hyperoxia-induced inflammatory acute lung injury can be attenuated by inhibition of extracellular HMGB1 [16]. In turn, IL-6 leads to higher STAT3 expression (including the stimulation of hSP-B transcription in respiratory epithelial cells) [37]. These cytokines promote naive T cells differentiation to Th-17, mediated by ROR gamma, STAT3 transcription factor [38] and HMGB1—an important promoter of Th17 cell differentiation through the elevation of ROR-γt mRNA expression [39].

### Antioxidant effect of exogenous surfactant exposure prior hyperoxia insult

The antioxidant effect of SP-B was observed in the present study, in vivo, when the levels of SOD, TBARS, and protein carbonyl returned to normal after exposure to exogenous surfactant on hyperoxia-induced by inflammation. A recent study showed that SP-B exhibits good scavenging activities on HO^−^·and O_2_^−^ [40]. We speculate that protection against hyperoxia-induced inflammatory acute lung injury is initiated by the ability of the surfactant to significantly reduce oxidant radicals. Without oxidative stress and lipid peroxidation, cell membranes remain intact and the primary immune barrier is maintained. In addition, normal levels of reactive oxygen species maintain cell regulation, without apoptosis and cell signaling directed toward lung inflammation [41–43]. In vitro studies showed, however, that both surfactants, endogenous and exogenous, may be subjected to oxidative inactivation [44] and the oxidative modification, and functional inactivation of SP-A is accentuated when SP-A underwent lipoperoxidation [45]. Further studies will be needed to assess the importance of this phenomenon.

### Limitations of exogenous surfactant administration in adult patients with ARDS

Despite a strong biological rationale and positive results in pediatric patients, surfactant therapy has failed to demonstrate a mortality benefit in adult patients with ARDS [3]. One potential explanation for the lack of positive findings may stem from a lack of surfactant reaching the distal airways and alveoli as a result of suboptimal instilled dose volume. In recent computational and airway modeling experiments [46], it has been suggested that adult conducting airways have almost 100-fold more surface airway compared to neonatal airways and therefore require considerable amounts of instilled surfactant to become fully coated. Only after saturation of the proximal conducting airways can the distal airways be reached. Further mechanistic support for suboptimal delivery could be explained by highly concentrated formulation of surfactant used in adult studies. The same computational modeling [46] suggests that highly concentrated surfactant could further increase viscosity, thereby preventing distal delivery as may have been the case in two unsuccessful controlled trials in adult patients with ARDS by Spragg et al. [47, 48]. Furthermore, although we did not directly measure the lung distribution of surfactant, our experimental model of exogenous surfactant administration was modeled after the study by Ganguly et al. [49]. They demonstrated that intranasally administered particles deposited in non-target lung locations were translocated to peripheral sites in the lung therapeutically after surfactant application [49].

On the other hand, new studies with KO animals (HMGB1^−/−^) and/or pharmacological antagonists might have reinforced mechanistic relationships over association between surfactant treatment and changes in biological biomarkers in our experimental model or the association of hyperoxia and mechanical ventilation. This model does not include mechanical ventilation which would be present in the clinical setting. However, this allowed us to specifically identify the effects of surfactant by itself.

## Conclusions

In summary, our study demonstrated that the administration of exogenous surfactant was able to reduce the inflammation and oxidative stress of the lungs induced by hyperoxia in our experimental model. Our results open new perspectives for research on the mechanism of exogenous surfactant in protecting the airway and lungs, in oxygen-rich lung microenvironment, against oxidative damage and aggravation of acute inflammation induced by hyperoxia.

## Abbreviations

ANOVA: Analysis of variance
ARDS: Acute respiratory distress syndrome
ASC: Animal Science Center
BALF: Bronchoalveolar lavage fluid
CAT: Catalase
CCL5: Chemokine (C-C motif) ligand 5
ES: Exogenous surfactant
HMGB1: High mobility group box protein 1
HO: Hydroxyl radical
IFN: Interferon
IL-10: Interleukin-10
IL-17: Interleukin-17
IL-6: Interleukin-6
MDA: Malondialdehyde
O_2_^−^: Superoxide radical
ROR-γt: Mutant transcription factor retinoic acid-related orphan receptor-gamma t
SOD: Superoxide dismutase
STAT3: Signal transducer and activator of transcription 3
TBARS: Thiobarbituric acid reactive substances
TNF: Tumor necrosis factor
VILI: Ventilator-induced lung injury
Vv [a]: Volume density of the alveolar space
Vv [sa]: Volume density of the alveolar septum
